# Comparative analysis of the gut microbiota of mice fed a diet supplemented with raw and cooked beef loin powder

**DOI:** 10.1038/s41598-021-90461-7

**Published:** 2021-06-01

**Authors:** Hye-Jin Kim, Dongwook Kim, Kwan-Woo Kim, Sang-Hoon Lee, Aera Jang

**Affiliations:** 1 Department of Applied Animal Science, College of Animal Life Science, Kangwon National University, Chuncheon, 24341 Korea; 2 Animal Genetic Resources Research Center, National Institute of Animal Science, RDA, Hamyang, 50000 Korea

**Keywords:** Biochemistry, Gastroenterology

## Abstract

We used 16S ribosomal RNA sequencing to evaluate changes in the gut microbiota of mice fed a diet supplemented with either raw or cooked beef loin powder for 9 weeks. Male BALB/c mice (n = 60) were randomly allocated to five groups: mice fed AIN-93G chow (CON), chow containing 5% (5RB) and 10% (10RB) raw beef loin powder, and chow containing 5% (5CB) and 10% (10CB) cooked beef loin powder. Dietary supplementation with both RB and CB increased the relative abundance of *Clostridiales* compared to the CON diet (*p* < 0.05). Mice fed 10RB showed a significantly higher relative abundance of Firmicutes (*p* = 0.018) and *Lactobacillus* (*p* = 0.001) than CON mice, and the ratio of Firmicutes/Bacteroidetes showed an increasing trend in the 10RB mice (*p* > 0.05). Mice fed 10CB showed a higher abundance of *Peptostreptococcaceae* and a lower abundance of *Desulfovibrionaceae* compared with the CON mice (*p* < 0.05). Genes for glycan biosynthesis, which result in short-chain fatty acid synthesis, were enriched in the CB mice compared to the RB mice, which was correlated to a high abundance of *Bacteroides*. Overall, dietary RB and CB changed the gut microbiota of mice (*p* < 0.05).

## Introduction

The human gastrointestinal tract harbors more than 100 trillion bacteria^1^. Gut bacteria play a crucial role in nutrition and host health^2^, and prevent pathogenic colonization by consuming available nutrients and producing bacteriocins and metabolites such as short-chain fatty acids (SCFA)^3^. However, certain gut bacteria have been linked to an increase in the incidence of insulin resistance, type 2 diabetes, inflammatory bowel disease (IBD), asthma, and various cancers^4,5^.

Over 90% of the gut microbiota are represented by four major microbial phyla in humans: Firmicutes, Bacteroidetes, Proteobacteria, and Actinobacteria^6^. Gut microbial profiles can be altered by changes in macronutrient composition^7^. For example, a high consumption of carbohydrates and simple sugars is associated with an increase in *Prevotella* (in phylum Bacteroidetes), as in the case of vegetarians^6^. In humans and mice, dietary animal protein and several amino acids in the diet increased the presence of Firmicutes and decreased Bacteroidetes in the gut microbiota^8,9^. Additionally, a high-fat diet reduces the proportions of Bacteroidetes and increases the proportions of Firmicutes and Proteobacteria in mice^10^.

Beef, as a type of red meat, contains important micronutrients and high biological value protein needed for maintaining good health throughout life^11^. Some studies show that, in mice, supplementation with lean beef alters the gut microbiota compared to non-meat protein supplementation^2,7,8,12^. It has been reported that dietary lean beef in colitis-induced mice caused intestinal inflammation and reduced microbial diversity and specific bacteria such as *Akkermansia muciniphila* and *Clostridium coccoides*^13^. However, beef is a rich source of both protein and fat, and there is limited research on the direct influence of whole red meat consumption on the gut microbiota.

The nutrients contained in beef are digested and absorbed through the gastrointestinal tract, whereas indigestible beef components enter the colon and are used for microbial metabolism. Thus, the digestibility of beef can change the compounds reaching the colon and alter the diversity of gut bacteria and metabolites^2^. Similarly, it has been reported that dietary low-digestible (raw) starch showed lower bacterial diversity, population, and Firmicutes/Bacteroidetes ratio than dietary high-digestible (cooked) starch in mice^12^. Beef is generally consumed in cooked form, however, people in many countries, such as Mongolia, Netherlands, Korea, and Japan, traditionally eat raw meat like steak tartare, Yukhoe, and beef sashimi^14^. Cooking increases the digestibility of beef and causes hardening of the meat fibers by denaturation of the protein^12^. Studies investigating whether raw and cooked beef consumption differently affects the diversity and composition of the gut microbiota are limited.

In this study, we evaluated the effect of dietary raw and cooked beef loin powder (RB and CB, respectively) on the change in the gut microbiota and physical condition of mice.

## Methods

### Animals and diets

**Figure 1:**
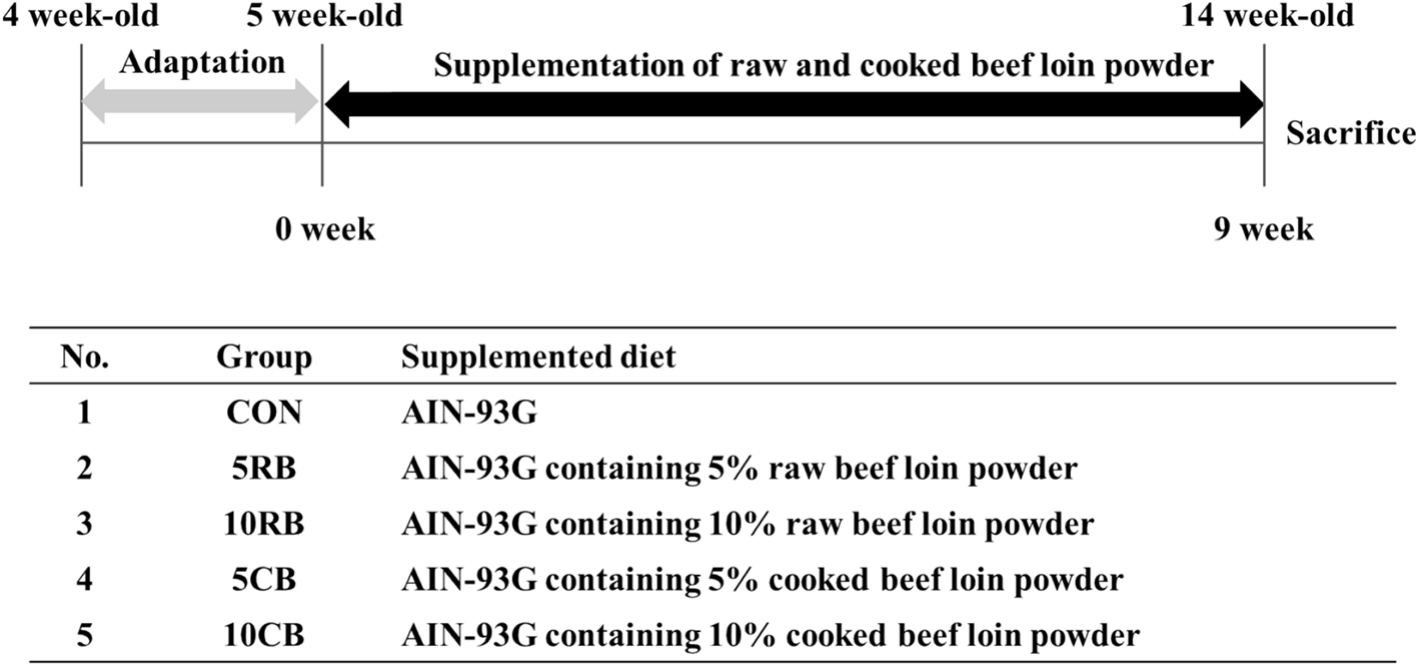
Experimental procedure and treatment group (n = 12/group).

A total of 60 male BALB/c mice (4 weeks old) were purchased from Orient Bio Inc. (Seongnam, Korea) and housed under standard conditions, with a controlled 12 h light–dark cycle, and 55 ± 5% and 22 ± 1 °C humidity and temperature, respectively. After 7 days of acclimatization, the mice were randomly assigned to 5 groups (Fig. 1). Fresh food was provided every two days. Water and feed were available ad libitum. Body weight was assessed weekly, and feed intake was measured once every two days.

To create the experimental diet, Hanwoo beef loin (Korean quality grade 1 +, *m. longissimus*) was purchased from a local meat shop in Korea, and the total aerobic bacteria quantity of the raw beef loin was 1.96 Log CFU/g (data not shown). Visible fat and connective tissue were removed. The raw beef loin was chopped and used for the RB diet. For generating the CB diet, the beef was placed into a plastic bag and boiled in a water bath until reaching an internal temperature of 75 °C for 45 min. Both raw and cooked beef loin were lyophilized with a freeze-drier and finely ground to make the experimental chow. The protein contents of the RB and CB were 41.34% and 41.39%, respectively, and the fat contents were 54.27% and 56.44%, respectively.

Animal diets containing RB and CB were prepared based on the American Institute of Nutrition guidelines according to the methods of Zhu et al.^8^, to maintain the nutritional requirements of growing mice (AIN-93G). The mice in group 1 (CON) were fed a standard diet (AIN-93G). The treatment diet was prepared by exchanging casein and soybean oil with beef protein and fat from RB and CB at 5% and 10% concentrations, maintaining the nutritional energy values (20% protein and 7% fat) (Supplementary Table S1). The 5% and 10% RB and CB supplementation were equivalent to 31.2 g/60 kg/day and 62.4 g/60 kg/day for humans, respectively, calculated as per the FDA guidelines^15^.

This study was approved by Kangwon National University’s Committee and was performed in accordance with Kangwon National University’s Committee on the Care and Use of Laboratory Animals Guidelines (KW-171127-3). This study was carried out in compliance with the ARRIVE guideline.

### Fecal sample collection and DNA extraction

After 9 weeks of feeding, before being sacrificed, a fecal sample was collected from each mouse (n = 12/group) and stored at − 80 °C until analysis. DNA was extracted from pooled samples (pooling three mice into one sample, with a total of four samples per one group) using a NUCLEOSPIN Soil kit (Macherey–Nagel, Düren, Germany) according to the manufacturer’s protocol.

### Amplification and sequencing

The 16S ribosomal RNA (rRNA) gene extracted from feces was amplified with Takara Ex-taq DNA polymerase (Takara Bio, Shiga, Japan) and universal primers^16^. The genomic DNA V4 region gene from the total extracted genomic DNA was used for amplification. The genomic DNA V4 region gene was amplified from the total extracted genomic DNA. Before sequencing, amplified genomic DNA was normalized to 50 ng per sample using a Spark 10 M multimode microplate reader (Tecan Group AG, Zurich, Switzerland). DNA sequencing was conducted by ILLUMINA. A DNA library was constructed by C&K Genomics and sequenced using the MiSeq platform (Illumina Inc., San Diego, CA, USA), which produced 2 × 250 bp paired-end products^17^.

### Data processing and statistical analysis

The microbial communities were analyzed using Quantitative Insights into Microbial Ecology (QIIME, http://qiime.org/index.html) version 1.9.1 software^18^. The raw sequence reads were demultiplexed and quality trimmed. Reads were clustered with operational taxonomic units (OTUs) by identified close-reference OUT picking with 97% similarity, using the GREENGENES 16S rRNA sequence database v13.8 as a reference^19^. OTUs were normalized to 14,000 reads per sample by single rarefaction. The microbial diversity of treatments was determined using alpha diversity (Shannon index) and richness (numbers of OTUs). The principal coordinate analysis (PCoA) on UniFrac distances showed the beta diversity, and was visualized with EMPEROR 3D visualization software^16^. Microbial abundance data were presented as mean ± standard deviation (SD). Differences among the physical conditions of mice, microbial diversity, and bacterial abundance among groups, were assessed by one-way analysis of variance (ANOVA) and Tukey’s HSD test for multiple mean comparisons, using the R (v3.5.0)^20^ at *p* < 0.05.

To predict functional and evolutionary genes from the microbiota, the biological observation matrix (BIOM) file, which included information of OTUs generated by QIIME, was compared to the Kyoto Encyclopedia of Genes and Genomes (KEGG) database^21^. Phylogenetic Investigation of Communities by Reconstruction of Unobserved States (PICRUSt), designed to predict metagenome functional content from genes, was used for the prediction of KEGG using normalized OTUs. We performed a linear discriminant analysis (LDA) effect size (LEfSe) analysis for statistical significance, features of each sample, and visualization^8^. We compiled microbial abundance tables with *p* < 0.05 and LDA > 2.0 using the web-based platform Galaxy for LEfSe analysis^21^.

## Results

### Physical condition of mice

The initial weight of the mice ranged from 20.70 to 21.35 g, with no significant difference between groups (Supplementary Table S2). At the end of the experiment (9 weeks), dietary RB and CB fed to mice did not significantly impact the final body weight, body weight gain, feed intake, and feed efficiency ratio (FER).

### Diversity and richness of the gut microbiota

**Figure 2:**
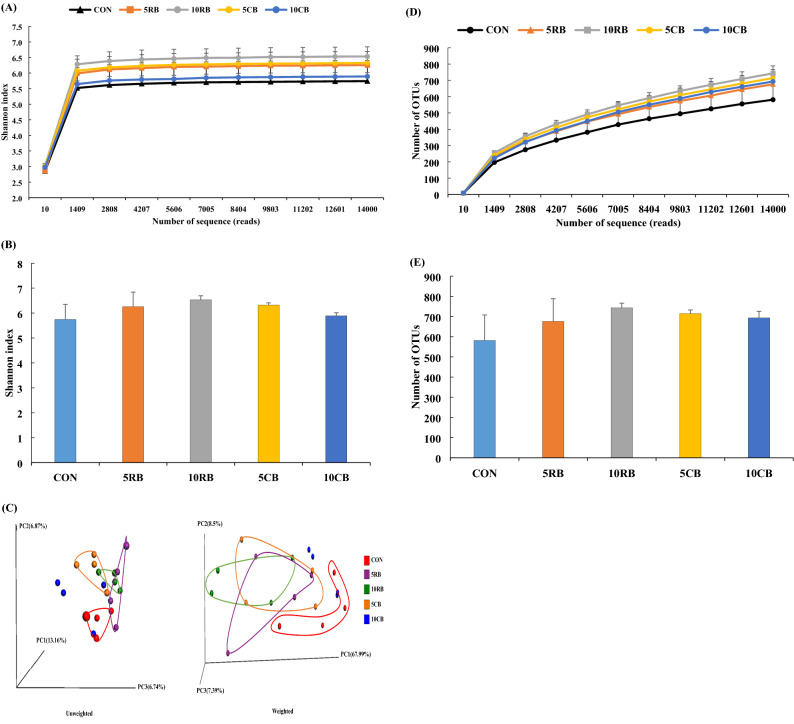
Effect of dietary raw and cooked beef loin powder on the richness and diversity of the microbiota (n = 12/group). Alpha diversity of the gut microbiota using Shannon index (**A**), differences of Shannon index at 14,000 reads (B), and the principal coordinate analysis of unweighted and weighted based on UniFrac distances (**C**). Rarefaction analysis for microbiota richness (**D**) and the number of OTUs in all groups at 14,000 reads (**E**). Data shown as the mean ± SD. One-way ANOVA with Tukey’s post-hoc test was used. CON, mice fed AIN-93G as chow; 5RB, mice fed AIN-93G containing 5% raw beef loin powder; 10RB, mice fed AIN-93G containing 10% raw beef loin powder; 5CB, mice fed AIN-93G containing 5% cooked beef loin powder; 10CB, mice fed AIN-93G containing 10% cooked beef loin powder.

The total number of OTUs was 3,407 in all experimental groups, with an average of 681 ± 61 per group. The alpha diversity was assessed using the Shannon index, which showed that estimates plateaued for all samples (Fig. 2A,B). Supplementation with RB and CB showed an increasing trend in the Shannon index, however, no statistical significance was observed (*p* > 0.05). The PCoA based on unweighted and weighted UniFrac distances is shown as beta diversity (Fig. 2C). There was no clear cluster among the treatments. Only the 10RB group showed a clearly separated cluster from the CON group at both unweighted and weighted levels. Supplementation with RB and CB tended to increase the number of OTUs compared to the CON group (Fig. 2D), however, no statistical significance was observed (Fig. 2E).

### Composition of gut bacteria

**Figure 3:**
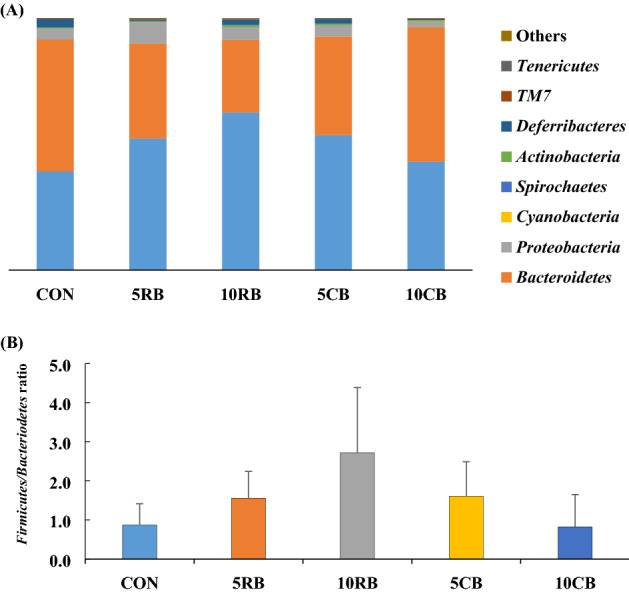
The composition of gut bacteria from BALB/c mice after supplementation with raw and cooked beef loin powder for 9 weeks (n = 12/group). Composition of gut bacteria of mice at the phylum levels (**A**) and the Firmucutes/Bacteriodetes ratio (**B**). Data shown as the mean ± SD. One-way ANOVA with Tukey’s post-hoc test was used. CON, mice fed AIN-93G as chow; 5RB, mice fed AIN-93G containing 5% raw beef loin powder; 10RB, mice fed AIN-93G containing 10% raw beef loin powder; 5CB, mice fed AIN-93G containing 5% cooked beef loin powder; 10CB, mice fed AIN-93G containing 10% cooked beef loin powder.

We detected 9 phyla, 26 classes, 48 orders, 106 families, and 203 genera in the gut microbiota community from fecal samples of mice fed RB and CB for 9 weeks. At the phylum level, Firmicutes and Bacteroidetes were major phyla in the gut bacteria of all groups (Fig. 3A). Cyanobacteria, Spirochaetes, Actinobacteria, TM7, and Tenericutes showed low abundance. In the CON group, Bacteroidetes (52.40%) was the predominant phylum followed by Firmicutes (39.14%), Proteobacteria (4.40%), and Deferribacteres (3.15%) (Table 1). The 10CB group had Bacteroidetes at 53.67% as the predominant phylum, and the 5RB, 10RB, and 5CB groups had Firmicutes (52.28–53.55%) as the major phylum. Notably, the proportion of Firmicutes in the 10RB group was higher than the CON group (*p* = 0.018). Aside from Firmicutes, there was no significant difference in phyla between all groups. Figure 3B shows the ratio of Firmicutes to Bacteroidetes (the F/B ratio) in mice after supplementation with RB and CB. The F/B ratio in the 5RB and 10RB groups tended to increase compared to the CON group, however, no significant difference was found (*p* > 0.05).

**Table 1:** Effect of dietary raw and cooked beef loin powder on the relative abundance of phyla in BALB/c mice (n = 12/group).

Phylum	CON	5RB	10RB	5CB	10CB	p value
*Firmicutes*	39.14 ± 7.371^b^	52.28 ± 5.556^ab^	62.57 ± 12.573^a^	53.55 ± 11.147^ab^	38.65 ± 13.611^ab^	0.018
*Bacteroidetes*	52.40 ± 8.069^a^	37.64 ± 12.517^a^	28.82 ± 13.048^a^	39.16 ± 14.531^a^	53.67 ± 16.949^a^	0.036
*Proteobacteria*	4.40 ± 2.607	8.52 ± 6.958	5.19 ± 1.977	4.51 ± 3.873	4.27 ± 2.735	0.290
*Cyanobacteria*	0.03 ± 0.018	0.05 ± 0.05	0.01 ± 0.007	0.03 ± 0.026	0.03 ± 0.027	0.310
*Spirochaetes*	0.03 ± 0.042	0.01 ± 0.015	0.01 ± 0.01	0.01 ± 0.009	0.02 ± 0.030	0.345
*Actinobacteria*	0.37 ± 0.162	0.40 ± 0.14	0.69 ± 0.332	0.67 ± 0.584	0.40 ± 0.191	0.485
*Deferribacteres*	3.15 ± 3.579	0.51 ± 0.419	2.16 ± 2.386	1.72 ± 2.913	2.36 ± 2.955	0.421
*TM7*	0.22 ± 0.200	0.31 ± 0.433	0.21 ± 0.145	0.16 ± 0.058	0.33 ± 0.249	0.901
*Tenericutes*	0.25 ± 0.171	0.28 ± 0.183	0.29 ± 0.266	0.18 ± 0.077	0.27 ± 0.197	0.909
Others	0.00 ± 0.005	0.01 ± 0.008	0.04 ± 0.056	0.01 ± 0.005	0.01 ± 0.017	0.442

Data shown as the mean ± SD. One-way ANOVA with Tukey’s post-hoc test was used. Within a row, different superscript letters indicate significant difference (*p* < 0.05). CON, mice fed AIN-93G as chow; 5RB, mice fed AIN-93G containing 5% raw beef loin powder; 10RB, mice fed AIN-93G containing 10% raw beef loin powder; 5CB, mice fed AIN-93G containing 5% cooked beef loin powder; 10CB, mice fed AIN-93G containing 10% cooked beef loin powder.

At the genus level, the 14 significantly different bacterial communities between the groups are shown in Table 2. An unclassified genus of the family *Peptostreptococcaceae* had a significantly higher relative abundance in RB and CB groups than the CON group (*p* = 0.001). *Lactobacillus* was the highest in the 10RB group compared to all other groups (*p* = 0.001). *Blautia* was significantly higher in relative abundance in the CB groups than the CON group (*p* = 0.010). Also, an unclassified genus of *Bacillaceae* had a higher relative abundance in the 5CB group compared to the CON group (*p* = 0.003). The genus *Bacteroides* in phylum Bacteroidetes was lower in the 10RB group than the CON group (*p* = 0.019); however, it increased in the 10CB group to a similar relative abundance as the CON group.

**Table 2:** Effect of dietary raw and cooked beef loin powder on the significant relative abundance of genera in BALB/c mice (n = 12/group).

Taxonomy	CON	5RB	10RB	5CB	10CB	p value
***Firmicutes***						
*o__Lactobacillales;f__Lactobacillaceae;* *g__Lactobacillus*	3.42 ± 3.374^b^	3.74 ± 2.295^b^	13.41 ± 4.88^a^	3.29 ± 2.008^b^	4.77 ± 1.186^b^	0.001
*o__Lactobacillales;f__Enterococcaceae;* *g__Enterococcus*	0.06 ± 0.054 ^b^	0.24 ± 0.137^a^	0.06 ± 0.043^b^	0.05 ± 0.028^b^	0.04 ± 0.008^b^	0.004
*o__Clostridiales;f__Veillonellaceae;* *g__Acidaminococcus*	0.25 ± 0.182^a^	0.05 ± 0.059^ab^	0.11 ± 0.082^ab^	0.02 ± 0.009^b^	0.04 ± 0.019^b^	0.025
*o__Clostridiales;f__Ruminococcaceae;* *g__Oscillospira*	3.56 ± 1.782^ab^	6.17 ± 2.318^ab^	6.95 ± 2.258^a^	5.04 ± 0.929^ab^	3.02 ± 0.638^b^	0.026
*o__Clostridiales;f__Peptostreptococcaceae;* *g__*	0.001 ± 0.002^c^	0.005 ± 0.004^bc^	0.014 ± 0.001^a^	0.012 ± 0.006^ab^	0.009 ± 0.003^ab^	0.001
*o__Clostridiales;f__Peptococcaceae;g__*	0.23 ± 0.138^ab^	0.37 ± 0.021^ab^	0.46 ± 0.152^a^	0.37 ± 0.130^ab^	0.18 ± 0.073^b^	0.017
*o__Clostridiales;f__Lachnospiraceae;* *g__Roseburia*	0.01 ± 0.004^a^	0.01 ± 0.004^a^	0.01 ± 0.005^a^	0.02 ± 0.009^a^	0.01 ± 0.006^a^	0.040
*o__Clostridiales;f__Lachnospiraceae;* *g__Blautia*	0.005 ± 0.007^b^	0.02 ± 0.01^ab^	0.009 ± 0.006^ab^	0.03 ± 0.002^a^	0.03 ± 0.019^a^	0.010
*o__Clostridiales;f__Lachnospiraceae;g__*	2.39 ± 0.903^b^	3.18 ± 1.396^ab^	5.28 ± 1.138^a^	4.44 ± 0.795^ab^	3.05 ± 0.647^b^	0.007
*o__Bacillales;f__Bacillaceae;g__*	0.01 ± 0.006^b^	0.01 ± 0.005^b^	0.09 ± 0.158^b^	0.37 ± 0.153^a^	0.24 ± 0.161^ab^	0.003
***Bacteroidetes***						
*o__Bacteroidales;f__Bacteroidaceae;* *g__Bacteroides*	30.66 ± 12.672^a^	16.42 ± 8.323^ab^	11.61 ± 7.395^b^	18.93 ± 7.782^ab^	30.89 ± 4.759^a^	0.019
*o__Bacteroidales;f__[Odoribacteraceae];* *g__Odoribacter*	0.90 ± 0.467^ab^	0.85 ± 0.557^ab^	0.59 ± 0.428^b^	0.91 ± 0.67^ab^	1.86 ± 0.545^a^	0.042
***Proteobacteria***						
*o__Enterobacteriales;f__Enterobacteriaceae;* *g__Proteus*	0.04 ± 0.022^a^	0.02 ± 0.018^ab^	0.01 ± 0.006^ab^	0.00 ± 0.002^b^	0.03 ± 0.015^ab^	0.025
*o__Desulfovibrionales;f__Desulfovibrionaceae; g__*	0.93 ± 0.635^a^	0.43 ± 0.121^ab^	0.70 ± 0.224^ab^	0.35 ± 0.19^ab^	0.23 ± 0.073^b^	0.043

Data shown as the mean ± SD. One-way ANOVA with Tukey’s post-hoc test was used. Within a row, different superscript letters indicate significant difference (*p* < 0.05). CON, mice fed AIN-93G as chow; 5RB, mice fed AIN-93G containing 5% raw beef loin powder; 10RB, mice fed AIN-93G containing 10% raw beef loin powder; 5CB, mice fed AIN-93G containing 5% cooked beef loin powder; 10CB, mice fed AIN-93G containing 10% cooked beef loin powder.

### Linear discriminant analysis of gut bacteria

**Figure 4:**
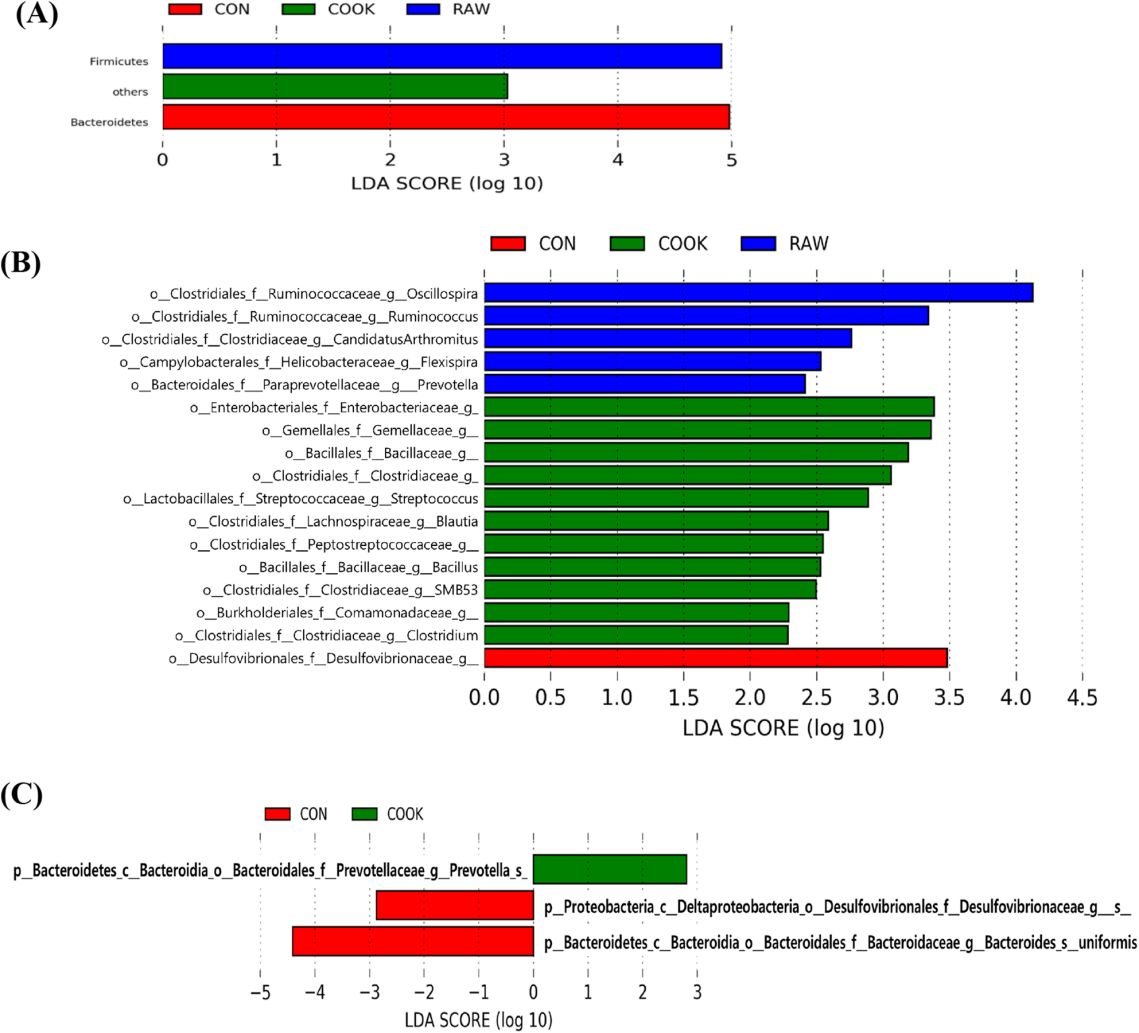
Comparison of microbial communities by supplementation with raw and cooked beef loin powder using Linear discriminant analysis (LDA) combined with effect size measurements (LEfSe) at phylum (**A**), genus (**B**), OTU (**C**) levels in BALB/c mice (n = 12/group). CON, mice fed AIN-93G as chow; RAW, mice fed AIN-93G containing 5% or 10% raw beef loin powder; COOK, mice fed AIN-93G containing 5% or 10% cooked beef loin powder.

To identify characteristic bacteria, we classified the original groups into three groups, including the CON, RAW (5RB and 10RB), and COOK (5CB and 10CB) groups, and performed LEfSe at each phylum, genus, and OTU level (Fig. 4). The phylum Firmicutes showed a high abundance in the RAW group, and the phylum Bacteroidetes was enriched in the CON group (*p* < 0.05, Fig. 4A). *Clostridia* (*Oscillospira, Ruminococcus, Candidatus* Arthromitus*), Flexispira,* and *Prevotella* were significantly enriched in the RAW group, while *Clostridia (Clostridiaceae, Blautia, Peptostreptococcaceae, SMB58,* and *Clostridium), Streptococcus*, and an unclassified genus of the family *Enterobacteriaceae, Gemellaceae, Bacillaceae,* and *Comamonadaceae*, were enriched in the COOK group (Fig. 4B). At OTU level (Fig. 4C), only three OTUs were significantly different among the groups. One of those OTUs was enriched in the COOK group, while the other two OTUs were high in the CON group. *Prevotella* was higher in the COOK group than the CON and RAW groups (*p* < 0.05)*. Desulfovibrionaceae* and *Bacteroides uniformis* were enriched in the CON group (*p* < 0.05)*.*

**Figure 5:**
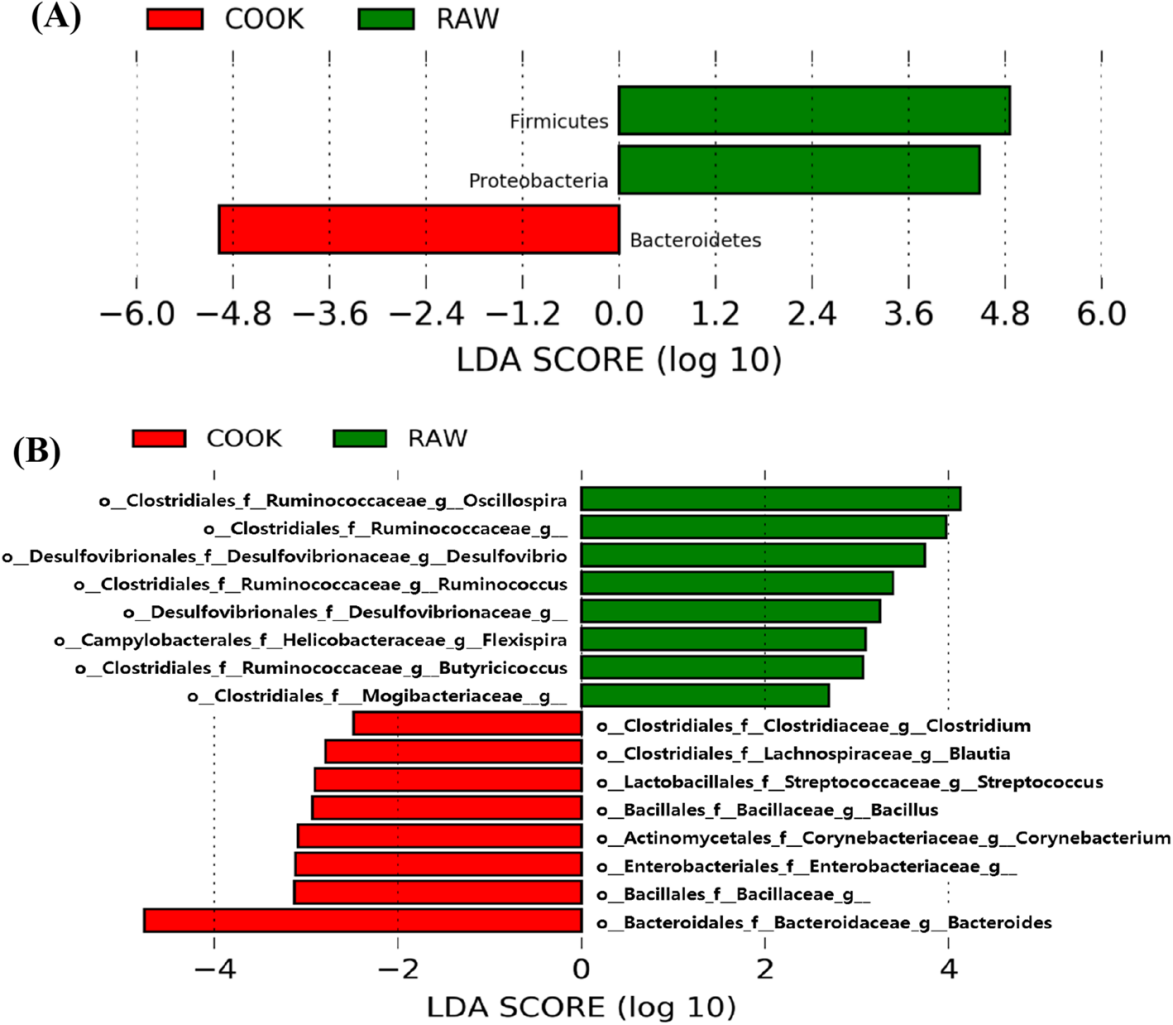
Comparison of microbial communities between supplementation with raw and cooked beef loin powder using linear discriminant analysis (LDA) combined with effect size measurements (LEfSe) at phylum (**A**) and genus (**B**) levels in BALB/c mice (n = 12/group). CON, mice fed AIN-93G as chow; RAW, mice fed AIN-93G containing 5% or 10% raw beef loin powder; COOK, mice fed AIN-93G containing 5% or 10% cooked beef loin powder.

When LDA analysis was performed between RAW and COOK groups, 3 phyla (Fig. 5A) and 16 genera (Fig. 5B) were significantly different. The RAW group had a higher abundance of the phyla Firmicutes and Proteobacteria than the COOK group, while the phylum Bacteroidetes was enriched in the COOK group (*p* < 0.05, Fig. 5A). Genera *Oscillospira, Desulfovibrio, Ruminococcus, Flexispira, Butyricicoccus,* and an unclassified genus of family *Ruminococcaceae, Desulfovibrionaceae, Mogibacteriaceae* were enriched in the RAW group (*p* < 0.05, Fig. 5B). However, the COOK group had higher abundance in the genera *Clostridium*, *Blautia, Streptococcus, Bacillus, Corynebacterium,* and *Bacteroides* and an unclassified genus of family *Bacillaceae,* and *Enterobacteriaceae,* than the RAW group (*p* < 0.05, Fig. 5B).

### Functional capacity of the microbial community

**Figure 6:**
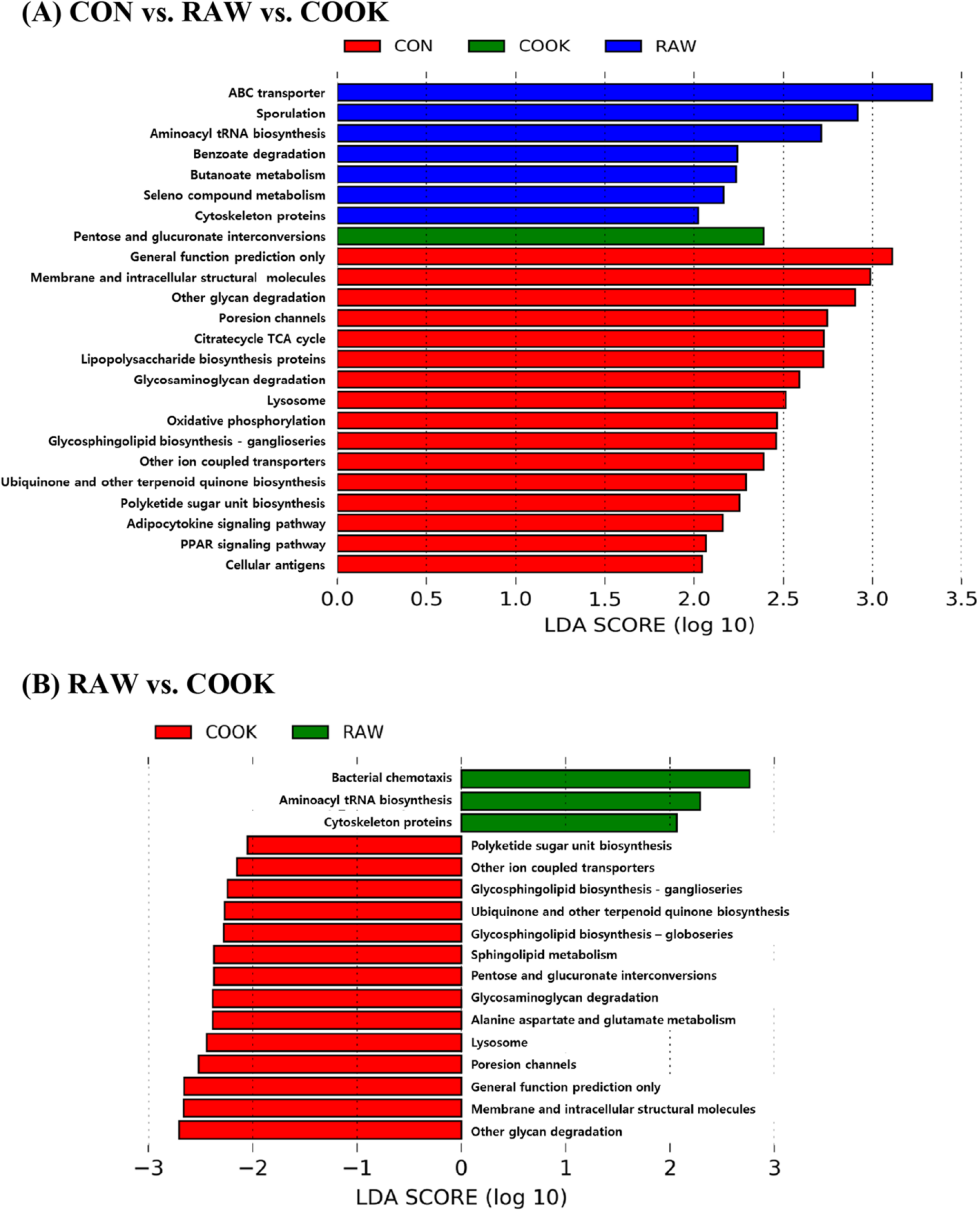
Functional capacity of the microbial communities associated with dietary raw and cooked beef loin powder using linear discriminant analysis (LDA) combined with effect size measurements (LEfSe) in KEGG (Kyoto Encyclopaedia of Genes and Genomes) pathways. RAW, mice fed AIN-93G containing 5% and 10% raw beef loin powder; COOK, mice fed AIN-93G containing 5% and 10% cooked beef loin powder.

Figure 6 shows the difference in KEGG categories of bacterial communities in the gut of CON, RAW, and COOK groups by LDA analysis. A total of 24 functional categories showed significant differences among groups (Fig. 6A). Seven functional categories were enriched in the RAW group, including ABC transporter, sporulation, aminoacyl tRNA biosynthesis, benzoate degradation, carbohydrate and amino acids metabolism (butanoate metabolism, seleno compound metabolism), and cytoskeleton proteins (*p* < 0.05). The pentose and glucuronate interconversions pathway was higher in the COOK group than the CON and RAW groups. Notably, the pathways concerned with carbohydrate metabolism, such as butanoate metabolism, pentose and glucuronate interconversions, were commonly increased in both RAW and COOK groups compared to the CON group (*p* < 0.05). The genes for glycan biosynthesis and metabolism pathways (other glycan degradation, glycosaminoglycan degradation, glycosphingolipid biosynthesis, and other ion coupled transporters), and the endocrine system (adipocytokine signaling and PPAR signaling pathway), were enriched in the CON group (*p* < 0.05). When the functional capacity of the gut microbials between the RAW and COOK groups was compared using LDA analysis, glycan biosynthesis and metabolism (other ion coupled transporters, glycosphingolipid biosynthesis, glycosaminoglycan degradation, and other glycan degradation) was identified as a predominant function in the COOK group compared to the RAW group (*p* < 0.05, Fig. 6B).

## Discussion

The gut microbiota is an important factor in the development and maintenance of the host immune response^1^. The gut microbiota can be changed by food, and plays a role between food and the host, including food digestion, fat metabolism, immune cell development and homeostasis, enteric nerve regulation, and epithelial homeostasis^22^. Moreover, the health effects of food on the host are highly associated with change in microbial diversity^23^. The diversity of the gut microbiota consists of alpha and beta diversity; the former represents species diversity within samples and the latter is the variation of species composition between samples^24^. High bacterial richness and diversity are related to good health^1^, and a reduction in microbial diversity can be linked to disorders of the intestine such as IBD and colorectal cancer^25^. After the diets of BALB/c mice were supplemented with RB and CB for 9 weeks, there was a trend of increasing alpha diversity (Shannon index) and richness (the number of OTUs) in both RB and CB groups of the gut microbiota compared to control mice (Fig. 2); however, no significant difference was found between the groups.

Changes in gut microbiota composition is related to dietary habits. Generally, Firmicutes has been linked to a high-fat diet^9^, and a high F/B ratio has been observed in obese individuals^26^ and relates to various health risks in the host; a high F/B ratio is generally associated with poor health^27^ and other metabolic disorders^16^. Also, increased *Lactobacillus* is present in mice fed a high-fat diet (60% fat, beef tallow)^28^. Zhu et al.^8^ reported that mice fed beef, pork, and chicken protein (20% protein in diet for 90 days) had a higher abundance of Firmicutes and *Lactobacillus* than mice fed non-meat protein. However, they reported that dietary beef, pork, and chicken did not increase the body weight of the mice, and the beef protein supplemented group had less body weight gain and visceral content than the non-meat protein supplemented group^8^. Similarly, we found an increased abundance in Firmicutes and *Lactobacillus* in the 10RB group compared to the CON group. However, dietary RB and CB did not lead to physiological changes in the mice, such as body weight gain and FER. This indicated that the increased proportion of Firmicutes by beef consumption does not necessarily lead to an increase in body weight, and further investigation into the relationship between beef consumption and the incidence of obesity remains necessary.

In the gut microbiota, there are some health-promoting bacteria, such as *Clostridiales, Lactobacillus, Bifidobacterium,* and *Bacteroides,* which help to maintain intestinal homeostasis. SCFA, such as acetate, propionate, and butyrate, are end-point compounds of fermentation by bacteria in the colon. They can be used as an energy source for colonocytes and impact colonic health^29^ and promote intestinal homeostasis^1^. *Clostridiales*, as one of the SCFA-producing bacteria, are commensal, gram-positive, rod-shaped bacteria in the phylum Firmicutes; this bacterium plays an important role in gut homeostasis by producing butyrate, which inhibits activation of the NF-κB pathway, leading to a decrease in pro-inflammatory cytokines^29^. In this study, *Clostridiales* were enriched in the gut microbiota of mice supplemented with both RB and CB compared to control mice (Fig. 4). However, the specific genus levels of *Clostridiales* were different between the RB and CB groups. First, supplementation with RB showed high amounts of *Oscillospira, Ruminococcus,* and *Candidatus* Arthromitus (Fig. 4). *Oscillospira* and *Ruminococcus* are butyrate producers and found in hosts supplemented with animal products^30^. *Candidatus* Arthromitus, as an important member of the mammalian gut microbiota, plays a crucial role in host immune function^31^. Second, supplementation with CB increased the proportion of *Clostridium*, *Blautia*, and *Peptostreptococcaceae* (Fig. 4)*. Clostridium* spp. and *Blautia* also produce butyric acid and acetic acid, and *Blautia* decreases obesity by regulating G-protein coupled receptors 41 and 43^32^. Moreover, a cohort study showed that harboring high amounts of *Blautia* was associated with reduced graft-versus-host disease lethality^33^. *Peptostreptococcaceae* are known as commensal bacteria, and are prevalent in the gut microbiota of healthy animals compared to diseased animals^34^, helping to maintain gut homeostasis.

There are also harmful bacteria, such as *Clostridium perfringens, Clostridium tetani, Enterobacteriaceae,* and *Streptococcus,* which induce intestinal dysbiosis and a poor gut environment by producing several toxins^6^. Some harmful bacteria were identified in the mice fed a diet supplemented with RB and CB, including *Streptococcus, Enterobacteriaceae*, and *Desulfovibrionaceae*. *Streptococcus* and *Enterobacteriaceae*, enriched in the dietary CB group compared to the dietary RB group (Fig. 5), reportedly increase in people with Crohn’s disease or anorexia nervosa^3^. The family *Desulfovibrionaceae* is associated with obesity and type 2 diabetes^3^, and was more prevalent in RB mice than in CB mice (Fig. 5).

In this study, supplementation with CB increased the relative abundance of *Bacillus* and *Bacteroides* (Fig. 5). *Bacillus* spp. have been isolated from healthy human gastrointestinal tracts, where they help other bacteria to adapt and colonize the area^25^. *Bacteroides* can also play a major role in SCFA production in the intestine^35^. In the present study, it attributed to a higher functional capacity for glycan biosynthesis and metabolism pathway in the CB group than in the RB group (Fig. 6). An increase in *Bacteroides* is related to intake of both meat protein and fat^7,36^. When lean meat powder (16%) was fed to C57BL/6J mice for 12 weeks, the amount of *Bacteroides* in the gut increased^7^. Bedani et al.^36^ similarly found that feeding dietary red meat (14% protein) and animal fat (40% fat) to mice for 60 days increased the amount of *Bacteroides* in the gut. In a human study, 380 g of supplemental cooked beef fed to males for 4 weeks resulted in a significant increase in the amount of *Bacteroides* in the gut compared to participants in the meatless diet^37^. The 5% and 10% of beef loin powder used in the present study were equivalent to the consumption of 31.2 g/60 kg body weight/day and 62.4 g/60 kg body weight/day for human, respectively. Beef protein and fat from the 5% RB and CB were 2% and 2.7–2.8%, respectively, while the 10% RB and CB were 4% and 5.4–5.6%, respectively. As the supplemented amount in this study was lower than the previously mentioned study, the 10CB mice did not show increased *Bacteroides* levels compared to the CON mice, however, the 10CB mice had increased *Bacteroides* levels compared to the 10RB mice (30.89% vs. 11.61%; Table 2). This could be attributed to the different intake amounts of fatty acids between the RB and CB mice. A high intake of monounsaturated fatty acids (MUFA) is associated with higher numbers of *Bacteroides* spp. in humans^6^ and mice^38^. Hanwoo beef loin reportedly contains a high proportion of MUFA compared to beef from Angus and Holstein^39^. The MUFA ratio in CB was higher than RB (41.05% vs. 38.57%). According to Patterson et al.^38^, a MUFA diet-fed mouse group (olive-oil containing 70.5% oleic acid) showed higher populations of *Bacteroides* than those fed a saturated fatty acid diet (palm oil containing 38.5% oleic acid) and n-3 polyunsaturated fatty acid diet (flaxseed/fish oil containing 10.8% oleic acid). Although the CB had a higher proportion of MUFA than the RB, it is not apparent whether the difference in MUFA contents changed the *Bacteroides* abundance in the gut of the mice, and this requires further investigation.

The mechanisms underlying the differences in the gut microbiota of mice fed a diet supplemented with RB and CB may come from the difference in digestibility. Food is digested in the stomach and absorbed in the small intestine, while undigested food fragments may enter the large intestine^8^. Approximately 10% of protein intake can reach the colon, which it is used as the main source of nitrogen for the growth of gut bacteria^40^, and can alter the diversity of gut bacteria^8^. Generally, animal proteins (dairy and meat proteins) have higher digestibility (over 90%) than plant proteins (70–90%)^41^. Moreover, the digestibility of protein in meat is higher in cooked meat than in raw meat^42,43^. In this study, the CB-supplemented diet (high digestibility) resulted in a higher number of Bacteroidetes in the gut microbiota than the RB-supplemented diet (low digestibility) (Fig. 5). Zhu et al.^2^ reported that the Bacteroidetes population was greater in mice fed chicken protein (high digestibility) than those fed beef and soy protein (low digestibility). Moreover, they reported that chicken protein showed higher digestibility and absorption in the small intestine than beef and soy protein, which resulted in lower amino acid contents in the colon.

In conclusion, supplementation with RB and CB induces changes in the bacterial composition of the gut microbiota in mice. Notably, mice fed the CB-supplemented diet showed a higher abundance of the phylum Bacteroidetes than mice fed the RB-supplemented diet. The genus *Bacteroides,* and some Clostridiales species, were of higher abundance in mice fed the CB-supplemented diet compared to mice fed the RB-supplemented diet, which resulted in an increase in the glycan biosynthesis and metabolism pathway in the CB-supplemented mice. These changes did not affect the physical condition of the mice. Although the difference in digestibility between RB and CB may contribute to changes in the gut microbial composition, as whole beef loin is a complex matrix of macromolecules, we are not able to discern a specific element responsible for the study results. Regardless, the findings broaden the understanding of the relationship between the gut microbiota and the consumption of raw and cooked beef loin.

## Supplementary Information

Supplementary Information.
