# Supplementary material for: Comparative analysis of the gut microbiota of mice fed a diet supplemented with raw and cooked beef loin powder
Source: Sci Rep. 2021 Jun 1;11:11489. doi: 10.1038/s41598-021-90461-7 (PMC8169908; doi:10.1038/s41598-021-90461-7)
Supplement: Supplementary file 1 — Supplementary Information. [file 41598_2021_90461_MOESM1_ESM.docx]

**Supplementary Information**

**Comparative analysis of the gut microbiota of mice fed a diet supplemented with raw and cooked beef loin powder**

Hye-Jin Kim^1^, Dongwook Kim^1^, Kwan-Woo Kim^2^, Sang-Hoon Lee^2^, Aera Jang^1,*^

^1^Department of Applied Animal Science, College of Animal Life Science, Kangwon National University, Chuncheon 24341, Korea

^2^Animal Genetic Resources Research Center, National Institute of Animal Science, RDA, Hamyang 50000, Korea

* e-mail: [ajang@kangwon.ac.kr](mailto:ajang@kangwon.ac.kr)

| Ingredients | Treatment (g/kg diet) | | | | |
| --- | --- | --- | --- | --- | --- |
|  | CON | 5RB | 10RB | 5CB | 10CB |
| Casein | 200.00 | 179.33 | 158.66 | 179.30 | 158.61 |
| Sucrose | 100.00 | 100.00 | 100.00 | 100.00 | 100.00 |
| Dextrose | 132.00 | 132.00 | 132.00 | 132.00 | 132.00 |
| Corn starch | 397.486 | 395.296 | 393.096 | 396.406 | 395.316 |
| Cellulose | 50.00 | 50.00 | 50.00 | 50.00 | 50.00 |
| Soybean oil | 70.00 | 42.86 | 15.73 | 41.78 | 13.56 |
| Raw Hanwoo beef loin powder | - | 50.00 | 100.00 | - | - |
| Cooked Hanwoo beef loin powder | - | - | - | 50.00 | 100.00 |
| TBHQ | 0.014 | 0.014 | 0.014 | 0.014 | 0.014 |
| AIN 93 Mineral mixture | 35 | 35 | 35 | 35 | 35 |
| AIN 93 Vitamin mix | 10 | 10 | 10 | 10 | 10 |
| L-Cystine | 3 | 3 | 3 | 3 | 3 |
| Choline bitartrate | 2.5 | 2.5 | 2.5 | 2.5 | 2.5 |
| Total energy value (kcal/g) | 4,000 | 3,982 | 3,991 | 3,991 | 3,996 |
| Carbohydrate (%) | 63.91 | 63.47 | 63.69 | 63.69 | 63.80 |
| Protein (%) | 20.3 | 20.3 | 20.3 | 20.3 | 20.3 |
| Fat (%) | 7 | 7 | 7 | 7 | 7 |

**Table S1.** The composition of animal diets containing raw and cooked beef loin powder (n=12/group). Total energy values were calculated using standard physiological fuel values for protein, carbohydrate, and fat, at 4, 4, and 9 kcal/g, respectively. CON, mice fed AIN-93G as chow; 5RB, mice fed AIN-93G containing 5% raw beef loin powder; 10RB, mice fed AIN-93G containing 10% raw beef loin powder; 5CB, mice fed AIN-93G containing 5% cooked beef loin powder; 10CB, mice fed AIN-93G containing 10% cooked beef loin powder.

| Treatment | Body weight (g) | | Body weight gain (g/day) | Feed intake (g/day) | Feed efficiency ratio (FER) |
| --- | --- | --- | --- | --- | --- |
|  | Initial | Final |  |  |  |
| CON | 21.35±0.414 | 28.67±1.046 | 0.12±0.012 | 3.17±0.117 | 0.04±0.004 |
| 5RB | 21.25±0.197 | 28.85±0.485 | 0.12±0.005 | 3.69±0.151 | 0.03±0.002 |
| 10RB | 21.03±0.680 | 28.20±0.438 | 0.12±0.005 | 3.39±0.246 | 0.03±0.003 |
| 5CB | 20.80±0.374 | 28.23±0.557 | 0.12±0.006 | 3.29±0.156 | 0.04±0.002 |
| 10CB | 20.70±0.438 | 28.23±0.596 | 0.12±0.004 | 3.12±0.116 | 0.04±0.002 |

**Table S2.** Effect of dietary raw and cooked beef loin powder on the body weight gain, feed intake, and feed efficiency ratio of BALB/c mice (n=12/group). Data shown as the mean ± SD. One way ANOVA with Tukey’s post-hoc test was used. CON, mice fed AIN-93G as chow; 5RB, mice fed AIN-93G containing 5% raw beef loin powder; 10RB, mice fed AIN-93G containing 10% raw beef loin powder; 5CB, mice fed AIN-93G containing 5% cooked beef loin powder; 10CB, mice fed AIN-93G containing 10% cooked beef loin powder.
